# Longitudinal Monitoring of Intra-Tumoural Heterogeneity Using Optical Barcoding of Patient-Derived Colorectal Tumour Models

**DOI:** 10.3390/cancers14030581

**Published:** 2022-01-24

**Authors:** Carolyn Shembrey, Jai Smith, Mélodie Grandin, Nathalia Williams, Hyun-Jung Cho, Christina Mølck, Corina Behrenbruch, Benjamin NJ. Thomson, Alexander G. Heriot, Delphine Merino, Frédéric Hollande

**Affiliations:** 1 Department of Clinical Pathology, University of Melbourne, Melbourne, VIC 3000, Australia; Carolyn.Shembrey@petermac.org (C.S.); jai.smith@unimelb.edu.au (J.S.); melodie.grandin@unimelb.edu.au (M.G.); Nathalia.Williams@unige.ch (N.W.); christina.moelck@gmail.com (C.M.); Cori.Behrenbruch@petermac.org (C.B.); 2 Victorian Comprehensive Cancer Centre, University of Melbourne Centre for Cancer Research, Melbourne, VIC 3000, Australia; 3 Biological Optical Microscopy Platform, University of Melbourne, Melbourne, VIC 3010, Australia; hcho@unimelb.edu.au; 4 The Sir Peter MacCallum Department of Oncology, University of Melbourne, Melbourne, VIC 3010, Australia; Alexander.Heriot@petermac.org; 5 Department of General Surgical Specialties, The Royal Melbourne Hospital, University of Melbourne, Melbourne, VIC 3050, Australia; Benjamin.Thomson@mh.org.au; 6 Department of Surgery, the Royal Melbourne Hospital, University of Melbourne, Melbourne, VIC 3050, Australia; 7 Department of Cancer Surgery, Peter MacCallum Cancer Centre, Melbourne, VIC 3000, Australia; 8 Department of Surgery, St Vincent’s Hospital, Melbourne, VIC 3065, Australia; 9 Olivia Newton-John Cancer Research Institute, Heidelberg, VIC 3084, Australia; delphine.merino@onjcri.org.au; 10 School of Cancer Medicine, La Trobe University, Melbourne, VIC 3086, Australia; 11 Immunology Division, The Walter and Eliza Hall Institute of Medical Research, Melbourne, VIC 3052, Australia; 12 Department of Medical Biology, The Faculty of Medicine, Dentistry and Health Science, University of Melbourne, Melbourne, VIC 3010, Australia

**Keywords:** organoids, tumour heterogeneity, colorectal neoplasms, clonal evolution, longitudinal imaging, neoplasm recurrence, metastasis, cell lineage, self-renewal, cell culture techniques

## Abstract

**Simple Summary:**

Colorectal cancer (CRC) is the second most common cancer worldwide. Despite improvements in the clinical management of CRC, outcomes for those with metastatic disease remain extremely poor. One reason for this is tumour heterogeneity, which refers to the observation that each cell within complex tumour cell populations displays different genetic features and biological behaviours. Such tumour heterogeneity is known to impact treatment efficacy and promote tumour recurrence. Here, we present a multi-colour barcoding methodology that allows for different lineages of colorectal cancer cells to be identified and monitored, thus allowing for tumour heterogeneity to be quantified in real-time. We show that discrete cell lineages can be quantified by both fluorescence microscopy and flow cytometry. Using this approach, we show that the cell culture models that are traditionally used in cancer research display limited heterogeneity, whereas patient-derived organoids—which are generated from fresh tumour resections—more faithfully represent the heterogeneity observed in cancer patients.

**Abstract:**

Geno- and phenotypic heterogeneity amongst cancer cell subpopulations are established drivers of treatment resistance and tumour recurrence. However, due to the technical difficulty associated with studying such intra-tumoural heterogeneity, this phenomenon is seldom interrogated in conventional cell culture models. Here, we employ a fluorescent lineage technique termed “optical barcoding” (OBC) to perform simultaneous longitudinal tracking of spatio-temporal fate in 64 patient-derived colorectal cancer subclones. To do so, patient-derived cancer cell lines and organoids were labelled with discrete combinations of reporter constructs, stably integrated into the genome and thus passed on from the founder cell to all its clonal descendants. This strategy enables the longitudinal monitoring of individual cell lineages based upon their unique optical barcodes. By designing a novel panel of six fluorescent proteins, the maximum theoretical subpopulation resolution of 64 discriminable subpopulations was achieved, greatly improving throughput compared with previous studies. We demonstrate that all subpopulations can be purified from complex clonal mixtures via flow cytometry, permitting the downstream isolation and analysis of any lineages of interest. Moreover, we outline an optimized imaging protocol that can be used to image optical barcodes in real-time, allowing for clonal dynamics to be resolved in live cells. In contrast with the limited intra-tumour heterogeneity observed in conventional 2D cell lines, the OBC technique was successfully used to quantify dynamic clonal expansions and contractions in 3D patient-derived organoids, which were previously demonstrated to better recapitulate the heterogeneity of their parental tumour material. In summary, we present OBC as a user-friendly, inexpensive, and high-throughput technique for monitoring intra-tumoural heterogeneity in in vitro cell culture models.

## 1. Introduction

Cancer is a highly heterogeneous disease whereby individual tumours and tumour cell subpopulations can display significant differences in their genetic, histopathologic, metabolic and immunologic profiles [1,2]. This inter- and intra-tumour heterogeneity is observed between tumours arising from different tissue or cell types, across patients with the same subtype of tumour, between primary and metastatic tumours within the same patient, and amongst individual cells from a single tumour. Indeed, tumours with a higher degree of clonal diversity have been shown to exhibit an enhanced capacity for metastatic progression [3,4,5,6,7] and treatment resistance [8,9], thereby conferring worse overall survival. This is notably the case in colorectal cancer (CRC) [10], the study model of the present work.

Lineage tracing is an approach used to assess intra-tumoural heterogeneity, and such clonal tracking can be adapted for both in vitro and in vivo settings. This suite of technologies (reviewed in [11]), in which the integration of a unique fluorescent or genetic barcode into individual cells enables their identification amongst heterogeneous cell pools, are powerful tools for addressing biologically relevant questions regarding clonal interactions, developmental trajectories, and the regulation of tissue homeostasis [12,13,14]. Lineage tracing is highly versatile and can be applied to virtually all cell types provided that three fundamental requirements are met: the lineage tracer should be passed on from founder cells to all clonal descendants, should be retained over time, and should not be transferable to unrelated or neighbouring cells. The major benefit of this approach is that it does not require prior definition of cellular markers of interest, allowing researchers to simultaneously evaluate various subpopulations without phenotypic bias. It was successfully used to monitor heterogeneity during the growth of CRC xenografts following transplantation in immunocompromised mice [15].

Previous genetic barcoding studies have revealed the enormous scope of intra-tumour heterogeneity, with reports of up to 1700 clones with distinct phenotypes and growth rates within a single tumour [16]. However, assessments of clonal contributions using this approach rely entirely upon the quantification of sequence reads, and the fidelity between barcode abundance and the resulting read counts has recently been questioned [17]. Furthermore, all sequencing methods face certain drawbacks, including errors associated with the amplification of small starting materials, challenging rates of false hits and a requirement for complex bioinformatics analysis. Most importantly, genomic data alone is unable to report on the functional importance of the cells interrogated, as endpoint assays preclude the continued monitoring of cells in culture. This caveat is particularly important when studying cellular interactions at the subpopulation level, as it limits the ability of researchers to determine whether the features which support the outgrowth of dominant clones are pre-existing or acquired de novo in response to selective pressures. For this reason, methods allowing for the longitudinal examination of clonal interactions in live tumour cell populations are desirable.

As compared with genetic barcoding, fluorescent barcoding approaches use multiple fluorescent proteins (FPs) to generate stable fluorescent signatures in recipient cells which can be visualized in real-time. Perhaps the most renowned example is the in vivo mapping of neuronal networks using transgenic Cre/lox *Brainbow* mice, in which site-specific recombination results in the stochastic expression of two, three or four fluorescent proteins [18,19]. *Brainbow* has been adapted for use in other model organisms, including drosophila melanogaster [20,21] and zebrafish [22,23], and it is possible to use the very similar Clonal Labelling of Neural Progenies (CLoNe) tool in all vertebrate species [24]. Moreover, to enable investigation into non-neural systems, the *Brainbow 2.1* cassette was modulated to produce the *R26R-Confetti* construct, which has been used for in vivo lineage tracing in various cell types, including glomerular [25], corneal [26] and Lgr5+ intestinal stem cells [27]. However, as the probes used in each of these methods exist on a continuum of fluorescence intensities, it is difficult to reproducibly purify these subpopulations using FACS, and thus, such approaches are primarily qualitative in nature and preclude the downstream analysis of any subpopulations of interest. There is, therefore, an unmet need for novel techniques that allow for both the in situ monitoring of individually tagged subpopulations as well as their isolation from complex polyclonal mixtures.

A novel approach to study heterogeneity is through optical barcoding (OBC), achieved by the simultaneous introduction of multiple reporter constructs per cell that each encode a different fluorescent protein (FP). The combinatorial expression of these FPs allows for multiple cell lineages to be traced based on their unique fluorescent signatures. Given the stable integration of these constructs into the genome of the target cell, these fluorescent signatures are passed on from the founder cell to all its clonal descendants, thus enabling the longitudinal monitoring of discrete subpopulations (hereby referred to as a “cluster”) over time. Importantly, as the number of FPs employed for barcoding is user-defined, and the throughput of this technique can be upscaled depending on the application of interest.

Early iterations of the OBC technique were based upon the additive red-green-blue (RGB) additive colour principle, in which the combinatorial expression of red, green and blue fluorescent proteins (mCherry, Venus and Cerulean, respectively) in heterogeneous cell populations can theoretically produce cellular clones with fluorescence signatures corresponding to all hues of the visible colour spectrum [28]. RGB marking has been implemented to monitor the clonality of liver regeneration following transplantation of RGB-barcoded primary hepatocytes [29], as well as to study the heterogeneous sensitivity of CRC cells to stress signals [30]. However, like the aforementioned techniques, these studies define individual subpopulations based on the fluorescent intensities of each vector and therefore make FACS-based quantitation difficult.

Recently, the OBC technique has been expanded from three to six fluorescent vectors [31], greatly expanding the number of subpopulations that can be unambiguously identified and traced within a single experiment. The major benefit of upscaling the number of fluorescent constructs used within an OBC panel is that the subpopulations can be defined in a binary manner (i.e., “blue” or “not blue”), thus circumventing the issues created by fluctuating fluorescent intensities.

Using this binary approach, recent studies have been able to trace clonal heterogeneity in breast cancer [32] and malignant glioma [31]. However, until now, optical barcoding has been limited to the quantitation of up to 41 subpopulations, using flow cytometry (FC) only. Imaging was used on smaller numbers of subclones (up to 31), limiting the spatial analysis of large sub-clonal interactions. In this study, we report a novel panel of FPs that allows for all theoretical subpopulations to be unambiguously discriminated by flow cytometry, thereby enhancing the resolution of the OBC technique. This panel is used in patient-derived colorectal cancer (CRC) cells grown in 2D as well as organoids grown in 3D. We also demonstrate for the first time that this optimized FP panel can expand the OBC approach to allow for live-cell imaging studies, thus permitting real-time monitoring of cellular responses in situ. Finally, by combining these approaches, we demonstrate that OBC can be used to trace and quantify dynamic clonal interactions in 3D patient-derived CRC organoids.

## 2. Results

### 2.1. Validation of OBC Conditions in Patient-Derived CRC Cells 

**Figure 1:**
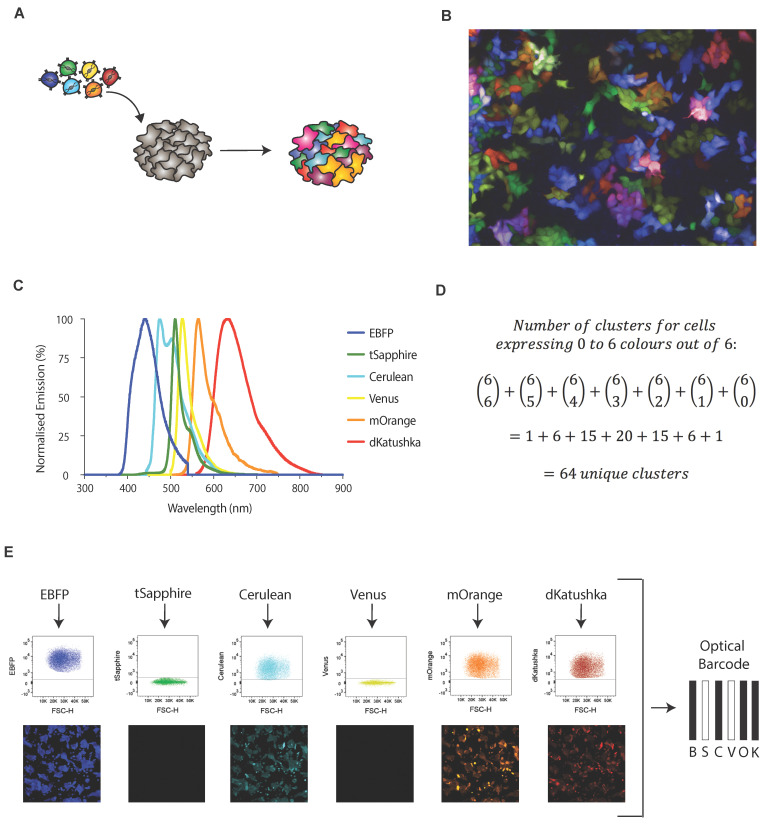
Optical barcoding as a tool to monitor intra-tumoural heterogeneity. (**A**) Optical barcoding is facilitated by the simultaneous transduction of heterogeneous cell populations with six different FPs, generating a multicolour pool of cells with unique fluorescent signatures. (**B**) Representative image of an optically barcoded cell line. (**C**) The six FPs employed in the optical barcoding panel are EBFP (B, blue), tSapphire (S, green), Cerulean (C, cyan), Venus (V, yellow), mOrange (O, Orange) and dKatushka (K, red). Curves show emission spectra only. (**D**) Using a binomial coefficient, 64 distinct barcodes will be generated when cells are transduced with 6 constructs and allowed to express 0 to 6 colours simultaneously (1 six-colour combination, 6 five-colour combinations, 15 four-colour combinations, etc.). (**E**) Expression levels for each FP are scored in a binary manner, generating a ‘barcode’ nomenclature that summarizes the fluorescent signature of each cluster. Solid bars denote FP-positivity, whereas blank bars indicate FP-negativity.

OBC is achieved via the simultaneous transduction of wild-type cells with several fluorescent constructs, thus generating a multi-coloured cell pool. As fluorescent signatures are passed on from the parental cells to all their clonal descendants, individual cellular subpopulations are identifiable based on their distinct fluorescent signature (Figure 1A,B). In our study, the OBC panel is composed of six different FPs (EBFP, blue; tSapphire, green; Cerulean, cyan; Venus, yellow; mOrange, orange; dKatushka, red) with unique excitation and emission spectra (Figure 1C). Thus, using a binomial coefficient, the concurrent expression of up to six of these colours should theoretically generate 64 clusters discriminable by their unique fluorescent signatures (Figure 1D). The proportion of each cluster is defined by the transduction efficiency for each of the six colours. At a transduction efficiency of 50% per colour, 64 subpopulations of equal size (each representing 1.56% of the total) should be generated. We observed that actual values deviate from this prediction, depending on the actual TE values obtained experimentally. In this study, the submaximal transduction efficiency of 40–70% was accepted, as this facilitates optimal colour diversity. Lower percentages result in a large proportion of indistinguishable, non-barcoded cells, whilst higher percentages increase the proportion of five and six vector positive cells, decreasing cluster diversity. 

It is well established that various cellular events such as cell division can modulate FP intensity in vitro. Although fluctuations in fluorescence can be exploited for quantitative imaging of both RNA biogenesis and protein–protein interactions [33], this property would be a hindrance in the context of lineage tracing as it may cause cells to “shift” from one lineage assignment to another based on their functional state. Thus, rather than enumerate fluorescent intensity for each probe, the OBC method quantifies the expression of each of the six FPs is annotated in a binary manner (i.e., “blue” or “not blue”) which, when combined, generates a fluorescent barcode that is less sensitive to transient changes in fluorescent signal. This feature ensures that clusters can be unambiguously identified and purified via FC, facilitating the cross-comparison of specific clusters throughout the course of a single experiment as well as across independent experiments. When expression levels are scored in this manner, fluorescent signatures can be described using a barcode nomenclature where solid and empty bars denote positivity and negativity for each FP, respectively (Figure 1E). 

### 2.2. OBC Does Not Alter Biological Properties of Wildtype Cells 

Although lentiviral transduction is a common tool employed in biomedical research, there is a small possibility that the introduced constructs may lead to insertional mutagenesis, resulting in genetic dysregulation at the site of viral integration. Such insertional mutagenesis can influence the composition of polyclonal populations, promoting the outgrowth of certain clones whilst suppressing others [34,35]. Although the LeGO vectors employed in this study are rendered self-inactivating (SIN) via a deletion in the 3′ LTR, which is known to lower the likelihood of insertional mutagenesis events, multiplexing six simultaneous transductions may conceivably result in off-target effects. Thus, to validate that the optically barcoded cells are representative of the parental cell lines from which they were derived, we tested the impact of optical barcoding on the proliferation, self-renewal properties and drug sensitivity of labelled CRC cell lines.

Three patient-derived CRC cell lines [36,37] were optically barcoded and their biological properties compared with that of their parental cell lines. The CPP14 and CPP35 lines were derived in our laboratory from treatment-naïve primary CRCs, whereas the CPP19 was generated from the liver metastasis of a patient treated with neo-adjuvant chemotherapy.

**Figure 2:**
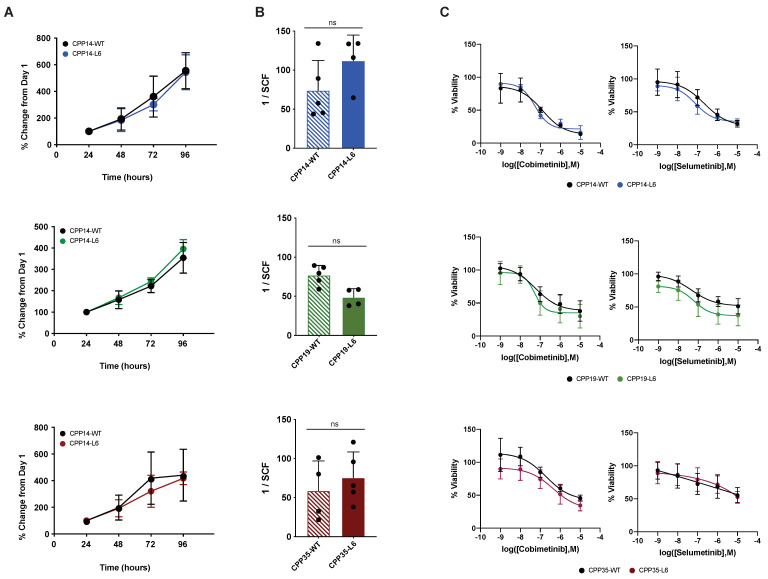
Optical barcoding does not perturb the biological properties of wild-type cells. Three patient-derived cell lines (CPP14, blue; CPP19, green; and CPP35, red) were optically barcoded (denoted by “L6” variants). (**A**) Proliferation was assessed via a resazurin metabolic assay performed at 24 h intervals for a period of 96 h (*n* = 4 independent experiments, mean ± SD). Doubling time (hours) ± 95% CI for the WT and L6 pairs were calculated by fitting an exponential growth equation (ns = non-significant, see Table S1). (**B**) ELDA performed to determine the SCF of the WT vs L6 cell pairs (*n* = 4 independent experiments, mean ± SD). The presence or absence of colonospheres was assessed 10 days after seeding at densities of 1000, 1000, 10 or 1 cells per well and is reported as the mean SCF ± SD (P = ns, Student’s t-test). (**C**) WT and L6 cells were treated with escalating doses of the MEK inhibitors cobimetinib and Selumetinib for 72 h, at which point cell viability was assessed via resazurin assay. Data is normalized to the vehicle control for each compound and reported as mean % viability ± SD (*n* > 4). LogIC50 ± 95% CI values were calculated by interpolating sigmoidal dose-response curves (see Table S2). WT, wild-type; ELDA, Extreme limiting dilution analysis; SCF, stem cell frequency.

Proliferation assays conducted over a 96 h period confirmed that there was no significant difference in growth kinetics nor doubling time between the parental (WT) and optically barcoded (L6) cells (Figure 2A; Table S1). Similarly, as a defining property of stem-like cancer cells is an increased capacity for self-renewal, we used Extreme Limiting Dilution Analysis (ELDA) [38] to infer the stem cell frequency (SCF) of the wild-type cell lines and their optically barcoded variants. This sphere-forming assay is designed for the enrichment of tumour-initiating cells and follows a single-hit hypothesis which postulates that only single cells endowed with self-renewal capacity should be able to generate spheres. In all three cell lines, there was no significant difference in SCF observed between WT and L6 pairings (Figure 2B). Lastly, to evaluate whether the OBC approach modulates therapeutic response, we compared the dose-response profiles of our overall WT and L6 cell populations after exposure to two targeted inhibitors of the MAPK pathway, cobimetinib and selumetinib. As the 95% confidence intervals for LogIC50 values were overlapping for all WT/L6 pairings, with LogIC50 fold changes ranging between 0.94 and 1.34, indicating that OBC did not significantly modify sensitivity to these compounds (Figure 2C; Table S2). Overall, these results indicated that the transduction of CRC cell lines with LeGO vectors didn’t affect their proliferation, self-renewal capacity and sensitivity to targeted inhibitors. 

### 2.3. A Novel FP Panel Allows for Enhanced OBC Resolution and Precise Barcode Quantification Using Flow Cytometry

**Figure 3:**
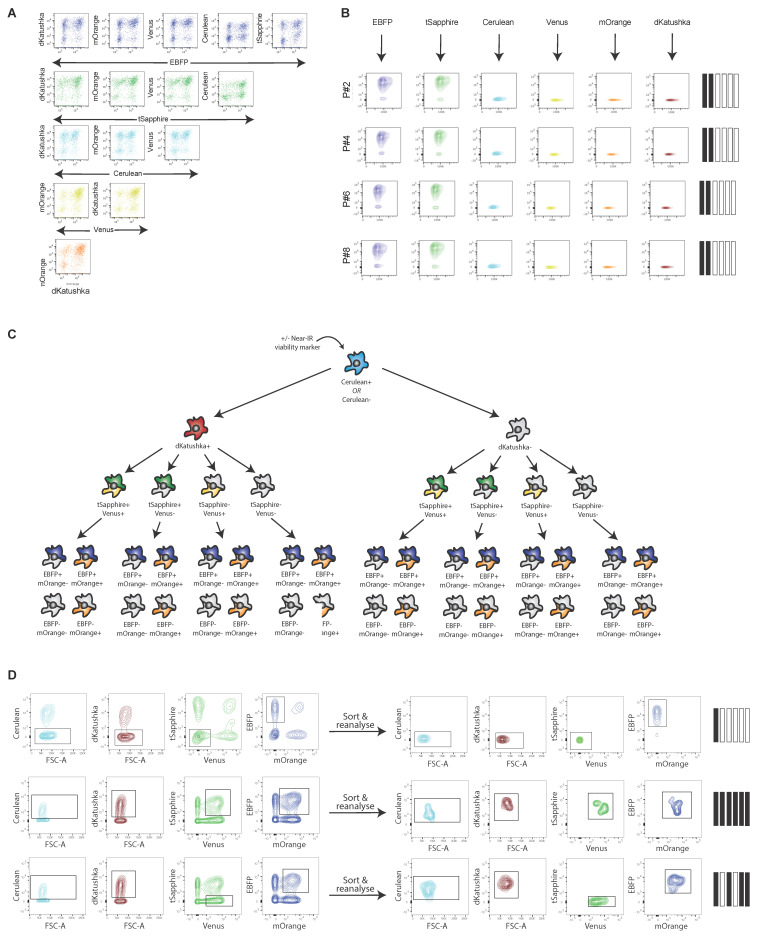
An optimized OBC panel allows for robust cluster separation via flow cytometry. (**A**) Bivariate compensation matrix demonstrates that all combinations of the six OBC constructs can be clearly distinguished using flow cytometry. (**B**) Barcoded subpopulations were isolated from the CPP35-L6 cell line and maintained in culture. Fluorescence signatures were analyzed by flow cytometry at each passage. Representative plots demonstrate the fluorescence stability of a single barcoded subpopulation for a duration of 8 passages (~4 weeks in culture). (**C**) A hierarchical gating strategy that combines sequential uni- and bivariate gates allow for each of the 64 barcoded subpopulations to be isolated via flow cytometry. Viability markers or antibodies are compatible with this strategy, provided they are conjugated to near-IR FPs. (**D**) Representative plots demonstrating that single barcoded populations can be purified from heterogeneous mixtures. Reanalysis of the sorted population confirms the accuracy of the gating strategy. IR, infra-red.

Next, we confirmed that our OBC panel performed robustly when analyzed by flow cytometry. Compensation matrices derived from this new panel demonstrates that all possible combinations of the six LeGO FPs can be clearly separated using FC (Figure 3A), including spectrally adjacent FPs (such as tSapphire/Cerulean). We next confirmed that the spectral barcodes assigned to different cell subpopulations were stable over time. Subpopulations with specific barcodes were sorted via FC, serially passaged in culture, and their fluorescence profiles were analyzed after every second passage. Over the course of one month and eight passages, no changes were observed in the barcode profiles of the barcoded population (Figure 3B).

We next asked whether a novel gating strategy that would allow for the purification of all 64 theoretical barcoded subpopulations could be designed. This would significantly increase the resolution of the OBC technique, which was previously restricted to the analysis of only 41 subpopulations. To this end, a hierarchical gating strategy was established (Figure 3C); here, the cells of interest are sequentially passed through a combination of univariate (i.e., Cerulean+ vs Cerulean-) and bivariate (i.e., EBFP+/mOrange-, EBFP+/mOrange+, EBFP-/mOrange+, EBFP-/mOrange-) gates encompassing each of the 6 FPs utilized in the OBC panel. As the near-infrared (near-IR) region of the visible spectrum is unoccupied, assessments of cell viability and/or other antibody applications can be performed upstream of this 6-colour deconvolution.

To test the fidelity of this gating strategy, heterogeneous mixtures of cells were purified into 64 uni-barcoded populations. Reanalysis of the sorted cells confirmed that the sorted populations maintained the expected fluorescence profile with no spectral leakage (Figure 3D), validating the precision of the gating and compensation settings. 

### 2.4. Spectral Imaging with Linear Unmixing Allows for Real-Time Tracing of Optically Barcoded Subpopulations

Although flow cytometry analysis is a powerful tool for the enumeration and purification of optically barcoded cells, it does mandate that the cells of interest be detached from cultureware or excised from recipient mice prior to analysis. As the in situ analysis of barcoded subpopulations would allow for additional morphological and spatial information to be acquired, we next asked whether live-cell imaging could be used for barcode quantification. 

**Figure 4:**
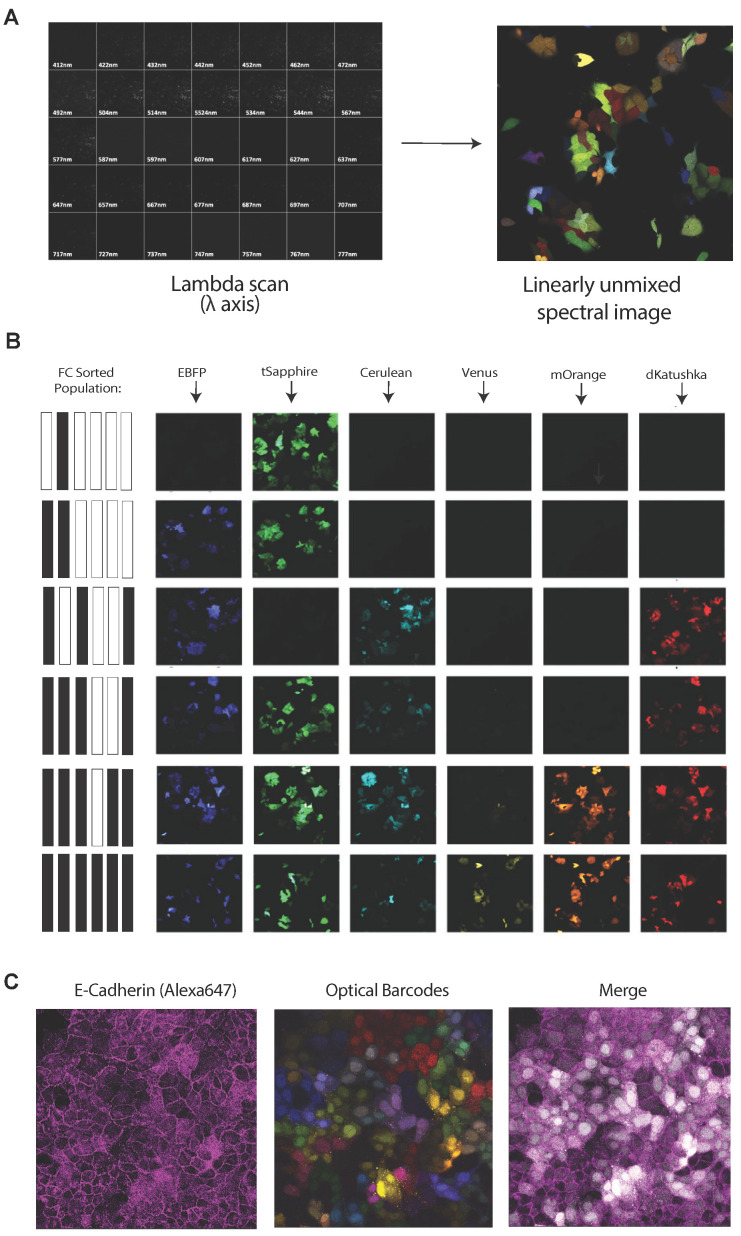
Spectral imaging with linear unmixing allows for real-time identification of optically barcoded subpopulations. (**A**) Schematic of the SILU analysis pipeline used to image optical barcodes in live cells. (**B**) Subpopulations with barcodes indicated at left were sorted, and their fluorescence signatures were corroborated via SILU. (**C**) CPP35-L6 cells were stained for E-cadherin to demonstrate the compatibility of far-red fluorochromes with the OBC panel, enabling seven-colour applications. In merged panel, OBC channels have been converted to greyscale. SILU, spectral imaging with linear unmixing.

To image optical barcodes in live cells, cells expressing known fluorescence barcodes were purified by FACS and subsequently imaged using spectral imaging with linear un-mixing (SILU) protocol (Figure 4A; see Methods for further details). Irrespective of the number of FPs present in the barcoded population (zero through six out of six), the fluorescence profiles obtained by SILU analysis were consistent with the ground-truth profiles obtained by flow cytometry (Figure 4B). Additionally, proof-of-concept staining with an AlexaFluor 647-conjugated antibody against E-cadherin revealed that the far-red region of the visible spectrum was compatible with spectral deconvolution using SILU (Figure 4C). Like the near-IR marker used for FC analysis, the addition of this 7th far-red probe allows for additional antibody-based studies to be multiplexed with the OBC panel, with the added benefit of being able to determine the cellular localization of the target of interest.

### 2.5. Applying OBC to Monitor Clonal Outgrowth in Heterogenous Tumour Cell Populations

Phylogenetic lineage-tracing studies have demonstrated that, over time, tumour cell populations may evolve in a manner that either supports the concomitant growth of multiple minor clones or promotes the emergence of a single, dominant clone [39]. Thus, having validated the technical aspects of the OBC system, we sought to determine whether OBC could be used to monitor differential growth kinetics at the subpopulation level. Preliminary analyses using conventional adherent CRC cell lines revealed limited shift in clonal composition over time (Figure S1), consistent with previous reports that the clonal diversity of cell lines grown as 2D culture models declines with increased passaging [40]. Subsequently, we elected to profile cluster dynamics in primary CRC cells grown as 3D structures termed patient-derived organoids (PDOs). By embedding patient-derived cells in a Matrigel matrix supplemented with physiologically relevant growth factors, PDOs adopt a 3D morphology that faithfully recapitulates the architecture of the parental tissue. The provision of ECM components like collagen and laminin not only supports 3D growth of these organoids but additionally mimics the physiological barriers which influence compound delivery and immune cell infiltration in in vivo lesions. Molecular and functional studies of large organoid biobanks have confirmed that organoids retain patient-specific mutation profiles and mimic clinical responses to a variety of anti-cancer compounds [41,42].

**Figure 5:**
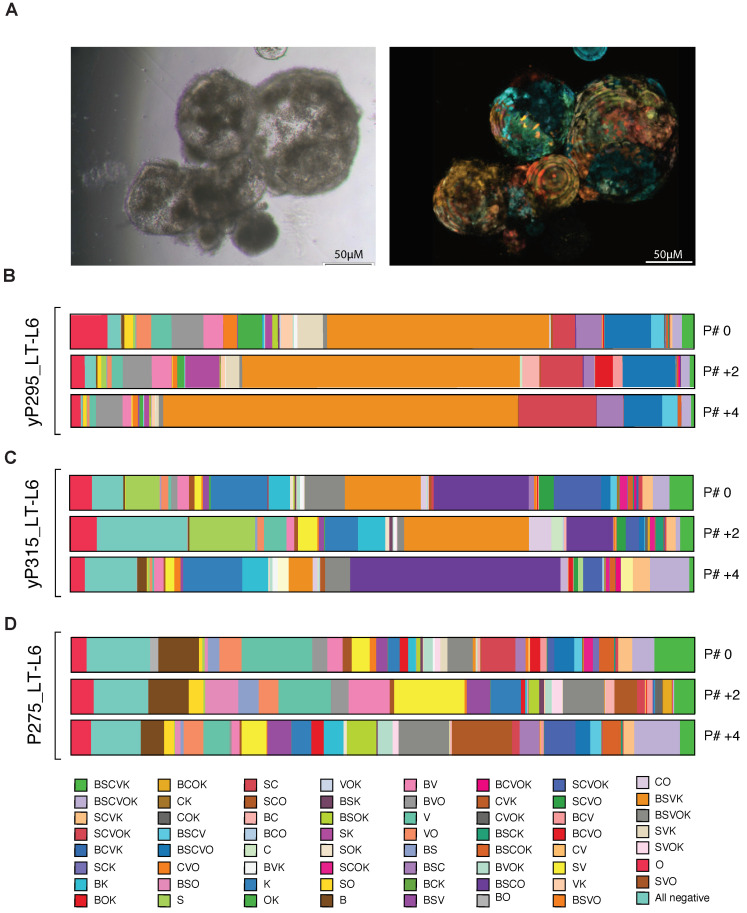
Optical barcoding of patient-derived organoids allows for the real-time estimation of subpopulation growth kinetics. (**A**) Representative brightfield (left) and fluorescence (right) images of optically barcoded CRC PDOs. (**B**–**D**) Bar-charts illustrating cluster frequencies following serial flow cytometric analysis of three optically barcoded PDO lines derived from primary colorectal liver metastasis samples. (**B**) yP295_LT-L6 (**C**) yP315_LT-L6 and (**D**) P275_LT-L6. Each of the 64 colours represents a single optically barcoded subpopulation, analyzed at baseline (P#0) and after 2 (P# +2) and 4 (P# +4) passages (~4 weeks total duration).

We utilized a panel of PDOs obtained from the liver metastases of three Stage IV CRC patients, recently developed and characterized in our group [43], including one treatment-naïve sample, P275_LT, and two neoadjuvant FOLFOX-treated samples, yP315_LT and yP295_LT. Each PDO was optically barcoded (P275_LT-L6, yP315_LT-L6 and yP295_LT-L6; Figure 5A) and subjected to serial flow cytometric analysis to enumerate cluster distribution after every second passage (approximately 2–3 week intervals, depending on the growth rate of each PDO).

The BSVK+ population (orange) was the most dominant subpopulation in the yP295_LT-L6 sample (Figure 5B), steadily expanding from 35.6% of the total population to at P#0 to 56.8% by P#+4. Similarly, the SC+ population (salmon) steadily increased throughout the analysis period, expanding ~3.5-fold from 3.6% at P#0 to 12.5% by P#+4. Collectively, these results suggest that serial passaging of the yP295_LT-L6 line leads to profound contraction of most subpopulations in favour of the steady outgrowth of the major clusters.

For yP315_LT-L6 (Figure 5C), whilst the BSVK+ cluster (orange) was dominant at P#0 (12.2% of total cells), it had contracted to 3.8% by P#+4. Of note, this contraction was accompanied by the outgrowth of the other major subpopulation in this line, BSCO (purple), from 15.3% at P#0 to 33.7% at P#+4, suggestive of clonal competition between these 2 subpopulations. 

Conversely, P275_LT-L6 (Figure 5D) demonstrated a comparative lack of inter-clonal competition. Few dominant clusters were observed, with the largest subpopulation at any time point, V+ (seafoam green), comprising only 11.2% of the total cell population at P#0 (later contracting to = 4.2% by P# +4). Moreover, only 3 subpopulations (V+, seafoam green; BSVOK, grey; and the all-negative, teal) had populations above 5% of the total cell number at 2 out of 3 timepoints. 

Collectively, these results demonstrate that the OBC technique can be used to monitor the growth kinetics of individual cellular lineages as well as the complex interplay between these subpopulations in real-time.

## 3. Discussion

The isolation and cloning of green fluorescent protein (GFP) from *Aequorea victoria* revolutionized biomedical research, and since then, the repertoire of FPs with unique absorption and emission spectra has greatly expanded [44]. Multiplexing of these FPs has spawned several colour-based fluorescent barcoding technologies, such as OBC, which allow for cellular lineage tracing in real-time. Here, individual cells and all their clonal descendants within the barcoded population express a unique fluorescent signature, facilitating the spatio-temporal monitoring of unique subpopulations and assessment of their divergent behaviours. A major benefit of the OBC approach is the unbiased nature of cluster labelling, which does not require prior knowledge of specific markers of interest. Additionally, as compared with other endpoint analyses such as genetic barcoding, the ability to monitor fluorescent barcodes in real-time allows for longitudinal, live-cell analyses to be conducted, as well as enabling the prospective identification and isolation of different clusters of interest for subsequent studies.

In this study, we present a novel panel of FPs which, using a purpose-built multiplexed FC gating strategy as well as a novel SILU image analysis pipeline, enabled the unambiguous identification and purification of all 64 possible OBC subpopulations. This greatly improves the resolution of OBC as first proposed by Mohme and colleagues [31], where only 41 subpopulations were discriminable via FC, and simultaneously allows for the spatial distribution of the various subpopulations to be visualized. Indeed, amenability to FC analysis is a significant bottleneck that frequently reduces the throughput of fluorescent reporter technologies. For example, a novel transgenic mouse model has recently been introduced whereby stochastic recombination of a Cre-reporter construct can generate cells with over 100 unique multicolour signatures [45]. Although this, in theory, greatly extends the colour repertoire reported in previous techniques such as the *Brainbow* [18] and *Confetti* [46] mice, due to technical limitations associated with FC analysis, the quantification was restricted to a maximum of 15 colours per animal. Similarly, Maetzig and colleagues [47] recently described a method of multiplexed fluorescent barcoding that can generate up to 26 different colours; however, only 12 of these were traced in vivo. Although the SILU-based analyzes may need to be revised for ex vivo or intravital colour deconvolution, the FC pipeline presented herein is suitable for the analysis of optically barcoded cells cultured in vitro as well as dissociated primary tumour material recovered from animal models. Thus, it is expected that the novel OBC panel presented in this work may facilitate higher-throughput lineage tracing studies both in vitro and in vivo.

Optically barcoding a panel of PDOs derived from colorectal liver metastases allowed for distinct subpopulation interaction dynamics to be observed. For two of the three PDOs profiled, yP295_LT-L6 and yP315_LT-L6, serial passaging led to the emergence of a small number of dominant subpopulations. This is consistent with previous studies investigating the clonal composition of metastases. Studies using genetic barcoding in PDX models of triple-negative breast cancer (TNBC) demonstrated that a small proportion of subclones present in the primary tumour were the major contributors of metastases [48,49]. Interestingly, these results were not mimicked in the 2D setting, where limited dynamic clonal interactions were observed.

The OBC technique presented herein could also extend beyond monitoring growth kinetics at steady state. For example, as the presence of drug-resistant cells is known to undermine treatment efficacy [50,51,52], future studies could determine whether the OBC technique could be implemented to identify, monitor, and characterize drug-resistant subpopulations with temporal and spatial resolution. In this respect, it is noteworthy that P275_LT-L6, the only chemo-naïve sample included in this study, showed a relatively stable clonal distribution pattern, whereas yP295_LT-L6 and yP315_LT-L6, which were both previously exposed to neoadjuvant chemotherapy, demonstrated profound clonal expansion and contraction upon serial passaging.

## 4. Methods

### 4.1. Cell Lines and Tissue Culture

The CPP14, CPP19 and CPP35 patient-derived colorectal cancer cells (CPPs) were prepared from fresh colorectal cancer biopsies under Human ethics agreement #2011-A01141-100 40 (Nimes University Hospital, France), as described in [36,37]. Cells were maintained in Dulbecco’s Modified Eagle’s Medium (DMEM; Lonza, #12-614F) supplemented with 10% fetal bovine serum (FBS) (Bovogen Biologicals), Glutamax (2 mM; Life Technologies, #35050-061) and penicillin-streptomycin (10.00 U/mL; Life Technologies, #15140-122) at 37 °C in a humidified atmosphere with 5% CO_2_.

### 4.2. Patient-Derived Organoid Culture

Colorectal liver metastases were collected from patients undergoing treatment at Peter MacCallum Cancer Centre and St Vincent’s Hospital, Melbourne, Australia. This study was approved by the Peter MacCallum Cancer Centre Human Research Ethics Committee (HREC/15/PMCC/112, project #15/169), and written consent was obtained from each patient according to National Health and Medical Research Council guidelines.

Tumour samples of approximately 0.25–1 cm^3^ were collected directly from the operating theatre and used for the establishment of PDOs, which were maintained in Advanced DMEM/F12 (#2634010, Thermo Fisher Scientific) supplemented with Glutamax (2 mM; Life Technologies, #35050-061), penicillin-streptomycin (10.00 U/mL; Life Technologies, #15140-122), recombinant human EGF (50 ng/mL, Miltenti Biotec, #130-097-749), Leu [15] human Gastrin I (1 μg/mL, Merck, # G9145), N-acetyl cysteine (1 mM, Merck, #A9165), B27 (2X; Life Technologies, #17504044), A83-01 (ALK inhibitor; 500 nM, Merck, #SML0788), SB202190 (p38/MAPK inhibitor; 10 nM, Miltenyi Biotec, #130-106-275) and YP-27632 (ROCK inhibitor; 10 μM, Abcam, #120129) and at 37 °C in a humidified atmosphere with 5% CO_2_.

Organoids were cultivated in 24-well plates and typically passaged every 5–7 days at a split ratio of 1:2–1:4. Media was aspirated, and each well was washed with ice-cold PBS. Growth-factor reduced Matrigel matrix (Corning, #356231) was detached from the cultureware by scratching with the tip of a p1000 pipette and contents of each well were transferred to a falcon tube containing 10 mL of ice-cold PBS. Following centrifugation at 1200 rpm for 5 min, pelleted organoids were dissociated by mechanical trituration such that large aggregates were broken into small (<100 um) fragments. Fragments were resuspended in the appropriate amount of Matrigel (as determined by split ratio) and plated by adding 40 µL of cell suspension per well. When single-cell suspensions were required for flow, pelleted organoids were resuspended in 1 mL of pre-warmed TrypLE Express and incubated for up to 1 h at 37 °C in a temperature-controlled orbital shaker. To correct for variation in protein concentration between Matrigel batches, all Matrigel was pre-diluted to a concentration of 8.7 mg/mL in ice-cold basal DMEM/-F12. Matrigel domes were incubated at 37 °C and allowed to solidify for 30–60 min. Following incubation, 500 µL of complete organoid media was added to each well.

### 4.3. Production of Viruses Used for Optical Barcoding

To generate viral particles for optical barcoding, HEK-293T packaging cells were transiently transfected using *CaCl_2_*. Packaging cells were seeded in complete DMEM in 10 cm dishes at 80% confluency and incubated overnight. Four hours prior to transfection, complete media was refreshed. A transfection cocktail composed of packaging vectors (pMDLg/pRRE [10 mg; Addgene #12251], pRSV-Rev [5 mg; Addgene #12253] and pMD2.G/VSVg [2 mg; Addgene #12259]) and target DNA (10 mg per LeGO vector; Table 1) was combined with CaCl_2_ (2 M). Whilst air was bubbled through the solution, the DNA-CaCl_2_ mix was then added to an equal volume of 2 ×HEPES buffered saline (HEPES 0.05 M, NaCl 0.28 M, Na_2_HPO_4_ 1.5 mM [pH 7]). The solution was allowed to incubate for 20 min at RT, mixed well, then added gently to packaging cells and incubated overnight at 37 °C and 5% CO_2_. The following day, DMEM media was refreshed, and for the next 48 h, lentivirus-containing supernatants were harvested once daily. The harvested media was passed through a 0.4 mm filter to exclude cells and cellular debris. Viral titers were determined via titration of the 6 supernatants (one per vector) on each target cell line, analyzed 5 days later by flow cytometry on a FACSAria Fusion (Becton Dickinson) with a custom optical configuration (described in Section 2.5).

### 4.4. Optical Barcoding of Primary Cell Lines and Organoids

Six third-generation HIV-1-derived self-inactivating lentiviral gene ontology (LeGO) vectors (Table 1) were used for stable transduction of target cells as described previously [51]. Briefly, target cell lines (CPP14, CPP19, CPP35) were plated in 1 mL complete DMEM in a 24-well plate at a density of 50,000 cells per well. The following day, various amounts (as determined by the viral titres) of all 6 LeGO viruses were combined with 1 mg/mL polybrene, and the resulting mixture was added dropwise to the target cells. After overnight incubation at 37 °C and 5% CO_2_, complete DMEM media was refreshed. To optically barcode patient-derived organoids, the above procedure was performed; however, ULA plates were used to prevent cell adherence, and, the morning after transduction, organoids were harvested and replated in Matrigel domes (as described in Section 4.2). For both cell lines and organoids, the transduction efficiencies for each fluorescent protein were analyzed 2 passages later by flow cytometry on a FACSAria Fusion (Becton Dickinson) with raw cytometry data processed using FlowJo software (v 10.4, Tree Star Incorporated). Samples were accepted for further experimentation if the 6 fluorescent proteins were each expressed by a submaximal proportion of the barcoded cells (40–70%).

### 4.5. Resazurin Reduction Assay

Resazurin sodium salt working solution (Sigma-Aldrich, #R7017-1G) was prepared by resuspension in PBS (pH 7.4) to a final concentration of 0.15 mg/mL. Working solutions were filter sterilized and stored at 4 °C in light-protected tubes. Adherent cells were harvested using trypsin and resuspended at a concentration of 50,000 cells/mL. Cells were seeded into clear 96-well plates at a density of 5000 cells per well (100 μL of cell suspension) and allowed to adhere for 24 h. For proliferation assays, 20 μL of resazurin working solution was added to a set of quintuplicate wells every 24 h for a duration of 96 h. After incubation at 37 °C for 2 h, the resazurin reduction outcome measure was obtained by subtracting background readings from the absorbance measurement at 560 nM using the EnSpire multimode plate reader (Perkin Elmer, #6055400). For cytotoxicity assays, cells were treated, in triplicate, with the MEK inhibitors cobimetinib (Selleckchem, #S8041) or selumetinib (Selleckchem, #S1008) at various concentrations. After 72 h, 20 μL of resazurin working solution was added to each well and allowed to incubate at 37 °C for 2 h prior to absorbance measurement. Concentration-response curves were generated using non-linear regression analysis to determine drug potency (logIC_50_). Pooled graphs were generated from the means of data, normalized to the vehicle controls, from each individual experiment.

### 4.6. Extreme Limiting Dilution Analysis (ELDA)

ELDA was used to evaluate the self-renewal capacity of individual tumour cell lines as described previously. Briefly, adherent cells were harvested using Accumax Cell Dissociating Solution (Innovative Cell Technologies) for 15 min at 37 °C and mechanically pipetted until a single-cell suspension was obtained. Cells were plated in 96-well ultra-low attachment (ULA) plates (Corning Life Sciences, #CLS3474) in 100 μL Advanced DMEM/F-12 (Gibco, #12634010) supplemented with Glutamax (2 mM, Life Technologies, #35050-061) D-glucose (10% (*v*/*v*); Sigma-Aldrich, #G7021), N-2 Supplement (1% (*v*/*v*); Thermo Fisher Scientific, #17502048), human recombinant insulin (0.2% (*v*/*v*); Sigma-Aldrich, #I0908), human recombinant EGF (0.02% (*v*/*v*); Miltenyi Biotec, #130-097-749) and human recombinant FGF (0.01% (*v*/*v*); Miltenyi Biotec, #130-093-841) at cell densities of 1000, 100, 10 and 1 cell(s) per well, reflecting the dynamic range of the sphere-forming frequency of adherent cell lines. Twelve replicates for each dilution were obtained for each of the four treatment groups. 50 μL Media was replenished after 7 days in culture to prevent wells from drying out.

After 11 days in culture, sphere formation was evaluated by scoring each well for the presence (+) or absence (−) of a sphere(s) in a binary manner. The fraction of responding cells obtained at each density was then analyzed using the ELDA Webtool made available by the Bioinformatics Division of the Walter and Eliza Hall Institute of Medical Research (http://bioinf.wehi.edu.au/software/elda/, accessed on 18 January 2022) to derive the stem cell frequency (SCF) with corresponding 95% confidence intervals (CIs) for all treatments groups tested. For individual experiments, pairwise differences in SCFs across treatment groups were assessed using a χ^2^ test. For collated data, statistical analysis was performed using one-way, unpaired ANOVA followed by the Bonferroni correction for multiple comparisons.

### 4.7. Flow Cytometry

For analysis of optically barcoded cells, a custom optical configuration was designed (Table 2). To ensure accurate spectral deconvolution of optically barcoded cells, single-colour positive control cells were used for instrument compensation for each independent experiment. When viability staining was required, cells were incubated with LIVE/DEAD™ Fixable Near-IR Dead Cell Stain (Thermo Fisher Scientific, #L34975) for 20 min on ice, protected from light.

For all flow cytometry analysis and cell sorting, single-cell suspensions were resuspended in FACS Buffer (2% FBS, 0.2% EDTA in PBS) and passed through a 35 µm nylon mesh cell strainer to exclude large aggregates. All cytometry was performed on a FACSAria^TM^ Fusion cytometer (Becton Dickinson). Raw cytometry data were analyzed using FlowJo software (Tree Star Incorporated, v 10.4)

### 4.8. Spectral Imaging with Linear Unmixing

Imaging of optically barcoded cells was performed using the Olympus FluoView™ FV3000 (Tokyo, Japan) confocal microscope using a 20× air objective (numerical aperture of 1.2). To generate reference spectra for each FP, the confocal lambda-scanning mode (xyλ) was used to acquire emitted light in 10 nm bandwidths (2 nm step size) of the visible spectrum from CPP35 cells transduced with a single fluorescent construct. These spectral traces were recorded using the 3 laser excitations wavelengths, which correspond to those used for flow cytometric analyzes (405 nm, 488 nm and 561 nm) to harmonize the 2 techniques. Spectral deconvolution and image processing was performed using the normal unmixing function with background correction.

### 4.9. Statistical Analyses

All statistical analyses were performed using GraphPad Prism software (GraphPad, v8.2.1). Data are expressed as mean ± standard deviation (SD), pooled from a minimum of 3 independent experiments. The minimum threshold for rejecting the null hypothesis was *p* < 0.05. For results where statistics are shown, Student’s t-test was used (unless otherwise indicated) and significance is denoted as: * = *p* < 0.05; ** = *p* < 0.01; *** = *p* < 0.001.

## 5. Conclusions

Collectively, this study demonstrates that the user-friendly and high-throughput technique of OBC can be used as a tool for monitoring intra-tumoural heterogeneity in 2D and 3D cell culture models. We extend previous works by combining both FC and live-cell SILU-based analyses, allowing for quantitative readouts of subpopulation distributions to be complemented with spatial and temporal resolution. We envision that this optimized OBC panel will be useful not only in studying intra-tumoural heterogeneity but for lineage tracing experiments in other biological contexts.

## Figures and Tables

**Table 1:** Constructs used for optical barcoding.

Fluorescent Protein	Plasmid Name	Plasmid ID (Addgene #)	Excitation (nm)	Emission (nm)
EBFP	LeGO-EBFP2	85213	360–400	410–480
tSapphire	LeGO-S2	85211	360–400	500–550
Cerulean	LeGO-Cer2	27338	410–430	460–500
Venus	LeGO-V2	27340	490–510	500–550
mOrange	LeGO-mOrange2	85211	520–550	560–630
dKatushka	LeGO-dKatushka2	85214	600–630	640–680

**Table 2:** Optical configuration of BD FACSAria Fusion used for flow cytometric analysis 1.

Fluorochrome	Laser Line	Long-Pass Filter	Band-Pass Filter	Detector
EBFP	405 nm	N/A	430/25	C
tSapphire	405 nm	505	510/21	A
Cerulean	405 nm	450	485/22	B
Venus	488 nm	525	543/23	A
mOrange	561 nm	570	582/15	B
dKatushka	561 nm	750	780/60	A

## Data Availability

The data presented in this study are available on request from the corresponding author.

## Supplementary Materials

The following are available online at https://www.mdpi.com/article/10.3390/cancers14030581/s1, Figure S1: Optical barcoding of 2D CRC cell lines show limited change in cluster distrubtion upon serial passaging; Table S1: Doubling time estimation of wild-type and optically barcoded cell lines; Table S2: Profiling of MEK-inhibitor responses in wild-type and optically barcoded cell lines.
