# Supplementary material for: Longitudinal Monitoring of Intra-Tumoural Heterogeneity Using Optical Barcoding of Patient-Derived Colorectal Tumour Models
Source: Cancers (Basel). 2022 Jan 24;14(3):581. doi: 10.3390/cancers14030581 (PMC8833441; doi:10.3390/cancers14030581)
Supplement: Supplementary file 1 [file cancers-14-00581-s001.zip › cancers-1543884-supplementary.pdf]

Supplementary Data

Table S1 | Doubling time estimation of wildtype and optically barcoded cell lines

Cell Line	Variant	Doubling time (hours) ± 95% CI
CPP14	WT	31.7 ± 8.4
	L6	29.8 ± 9.6
CPP19	WT	39.7 ± 8.7
	L6	36.9 ± 4.9
CPP35	WT	40.3 ± 15.9
	L6	39.5 ± 12.05

Table S2 | Profiling of MEK-inhibitor responses in wildtype and optically barcoded cell lines

Cell Line	Variant	Drug	$LogIC_{50}$ ± 95% CI	Fold Change
CPP14	WT	Cobimetinib	-6.90 ± 0.56	0.95
	L6		-7.28 ± 0.30	
	WT	Selumetinib	-6.66 ± 0.62	0.94
	L6		-7.10 ± 0.46	
CPP19	WT	Cobimetinib	-7.24 ± 0.54	0.99
	L6		-7.29 ± 0.63	
	WT	Selumetinib	-7.33 ± 0.6	1.02
	L6		-7.22 ± 0.72	
CPP35	WT	Cobimetinib	-6.70 ± 0.84	1.06
	L6		-6.35 ± 0.98	
	WT	Selumetinib	-5.69^	1.34
	L6		-4.23^	

^ 95% CI unable to be determined as 50% cell death not achieved at this dose range

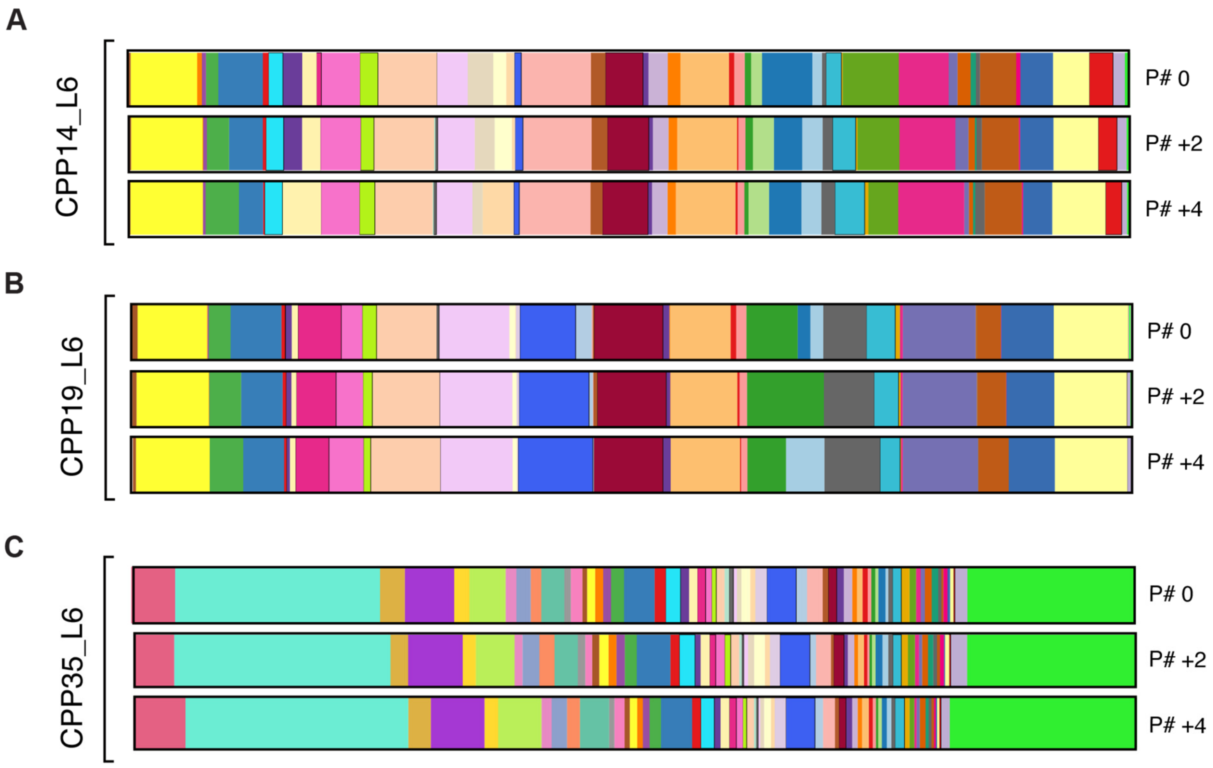

Figure S1 | Optical barcoding of 2D CRC cell lines show limited change in cluster distrubtion upon serial passaging

Barcharts illustrating cluster frequencies following serial flow cytometric analysis of three optically barcoded CRC cell lines; **(A)** CPP14-L6 **(B)** CPP19-L6 and **(C)** CPP35-L6.
